# Notes and News

**Published:** 1887-02-05

**Authors:** 


### NOTES AND NEWS.

COLLECTED AND COMMUNICATED.

THE Marquis of Ripon has kindly promised to preside at the annual festival dinner in aid of the Hospital for Sick Children, Great Ormond Street, at Willis's Rooms. The dinner has been fixed for Thursday 17th March.——The Governors of Guy's have not yet obtained quite £40,000 in response to their appeal to the public for £100,000. The Mercers' Company have contributed 1,000 guineas and Mr. Anthony Gibbs, £500. ——The Committee of the Cardiff Infirmary are going to take advantage of the Jubilee year to endeavour to clear off the debt, now amounting to about £7,000, on the new building. A children's ward is in course of erection, in pursuance of a legacy of £4,000 left to the Infirmary by the late Miss Shand.——Principal Paterson, of the Dundee University College, has announced the receipt of a donation of £6,000 towards a fund for establishing a Medical School in Dundee.——The whole of the proceeds of Mrs. Fawcett's recent lecture at Clapton on the "Social Progress of Women during the past 100 years," have been handed over by her to the New Hospital for Women, Marylebone Road.

IN view of the munificent donation of £2,000, left by the late Mr. John Barber to the City of Dublin Hospital, the directors have decided to name one of the wards of the institution the "Barber Ward." A similar bequest has been made by the deceased gentleman to the Mercer's Hospital, Dublin.

THANKS to the munificence of Mr. J. C. Clarke, the historic little town of Abingdon has been provided with a capital Cottage Hospital, containing eight beds and an excellent suite of offices. A sum of over £2,000 has been raised in the town, the interest on which will form the nucleus of an endowment fund.

THE Duke of Cambridge has kindly consented to take the chair at the 42nd annual festival in aid of the German Hospital, to be held at the Hotel Métropole on April 27th. This institution, which is very efficiently managed, is liberally supported by the Germans resident in London, and by the benevolent generally. Last year, 1,663 in-cases were admitted, while the out-patients attending the hospital and the two dispensaries in connection with it, numbered 23,219. Baron von Schröder, in presiding at the annual meeting the other day, described the present position of the German Hospital in London as "second to none."

THE Metropolitan Convalescent Institution, which was established in 1840, for the purpose of receiving poor convalescing patients from the London hospitals and dispensaries, to complete their restoration to health before returning to their own homes, has just completed a year of very successful work. The institution has now three Homes, the Asylum at Walton-on-the-Thames, a children's branch at Kingston Hill, and a seaside branch at Bexhill, near Hastings. In these Homes last year, 4,725 poor convalescents were received, free of all charge, and most of them returned very greatly improved. It is to be regretted that the funds of this excellent institution are not so flourishing as they deserve to be.

ACCORDING to the *Anglo-American Times*, "Hospital Saturday" has found its way to New York, where the promoters are asking for the ladies to exercise their "unlimited influence" in stimulating and extending sympathy and assistance for the sick and suffering poor.

The following vacancies are announced this week, the amount of the salary being placed in each case after the name of the institution :—

**Honorary Medical Officers.**

St. Bartholomew's Hospital. Assistant Physician.
Great Northern Central Hospital. Aural Surgeon. F.R.C.S.E

**Resident Medical Officers.**

Hereford Infirmary. Dispenser.—£40.
Liverpool Infirmary for Children. Assistant House Surgeon. —Board, etc.; no salary.
Liverpool Northern Hospital. Assistant House Surgeon.—£70.
Norfolk and Norwich Hospital. Assistant to House Surgeon. —Board ; no salary.

**Matrons.**

Great Northern Central Hospital.—£60, with board, etc.
Launceston Infirmary. £30, with board, etc.

**Trained Nurses.**

Essex and Colchester Hospital. For Male Surgical Ward.—£25.
Newport Infirmary, Mon. Charge Nurse.—£25, with uniform.

**Probationers.**

Newport Infirmary, Mon. Two.

---

Hospital Sunday in the metropolis has been fixed for June 19. Thus far the clergy, both of the Established Church and of the Non-Conforming bodies, have made a very general response to the circular appeal which is now being issued by the Council of the Fund, inviting their assistance by making collections in their respective churches and chapels. The Council intend using special efforts to make the Hospital Sunday Fund Collections in the present Jubilee Year worthy of the occasion and of the greatest city in the empire. For this purpose a sub-committee have been appointed, consisting of the Lord Mayor and Sir Sydney Waterlow, president and vice-president of the Fund ; Sir E. H. Currie and Mr. R. B. Martin, the honorary secretaries ; Sir Andrew Clark, Bart., M.D., president, and Mr. Henry C. Burdett, the Chairman of the Executive Committee of the Hospitals Association, the Hon. Reginald Capel, the Rev. Canon Fleming, and the Rev. Dr. Kennedy.

---

One of the oldest dispensaries in London is the Finsbury, which was established as far back as 1780. Last year no less than 15,912 new cases were dealt with, while 3,151 poor patients were visited at their own homes. From Hoxton, Shoreditch, Haggerston, and other neighbouring parishes, the poor now come in considerable numbers. In 1871, a gentleman left the sum of £10,000 for the establishment of a soup-kitchen and cottage hospital at Shoreditch, but thus far no site has been found. The Committee of the Finsbury Dispensary therefore think that, when this building is erected, it might be provided with waiting and consulting-rooms, and surgery, which could be used on three or four days a week for dispensary purposes.

---

Dr. Robert Macpherson recently writing to a contemporary on the abuse of medical charities, by the non-pauper public, related an incident where a "guid woman" who attended Anderson's College Dispensary, evinced charming candour when interrogated by the doctor as to her circumstances. "Why do you come here," he inquired of the would-be patient, "when you can pay a doctor?" "Oh," replied the canny dame, "I'm no gaun tae pay a doctor when I can get ane here for naething."

---

Lord Chelmsford draws our attention to an association, of which he is the honorary treasurer, the object of which is to provide a series of wholesome and nutritious dinners to poor convalescents upon taking their discharge from the hospitals. The period of convalescence is a critical time with the poor. The good food which they have been receiving in the hospital suddenly ceases, and their slender means, often none at all, prevent their having that nourishment which is just then so important for the full restoration of their health. Every case, before relief is granted, is submitted to full enquiry, and, if found deserving, the convalescent receives an order for a series of dinners, invariably given from a coffee-tavern in the neighbourhood of the recipient. At the present time the Convalescent Dinner Society are distributing orders for some 400 dinners a week, but this number falls far short of the number of deserving cases which would be dealt with if funds were more plentiful. Last year ninety-two-and-a-half per cent. of the amount received by the Society was expended by the Committee in providing dinners, the remaining seven-and-a-half per cent. being expended upon stationery, printing, and postage. The address of the hon. treasurer of the Society is 5, Knaresborough Place, South Kensington.

---

The Royal Maternity Charity, whose offices are at 31, Finsbury Square, E.C., has just completed the 130th year of its good and useful work. The charity was instituted in 1757 for delivering poor married women at their own homes. Last year the midwives attended just 4,000 cases, the highest number, excepting the year 1829, on record. Of the 4,048 children born, 3,894 survive, while there were only thirteen deaths among the mothers. Child-bearing is, as Sir John Lubbock described it at the recent annual meeting, always a painful and dangerous period, but in the case of the poor, where necessary comforts are not obtainable, it is much more so. The distinctive feature of the Royal Maternity Charity, is that it relieves poor married women without requiring their removal to the wards of a lying-in hospital, a very great advantage when we remember the importance of the constant presence of the poor mother in her home. The work of the Charity, moreover, is not circumscribed by walls, for, if sufficient funds were forthcoming, the words of its president, the Duke of Argyll, might be realised, "all London is the Charity's hospital, and every street a ward."

---

There seems to be little doubt that the friends of the Taunton and Somerset Hospital will succeed in their endeavour to raise £10,000, as a Jubilee Fund, to be appropriated for the permanent benefit of that institution. The hospital is one of the very best in England and confers its benefits not only upon the poor of Taunton, but upon those throughout West Somerset generally. The institution is just seventy-seven years of age, having been founded, it is interesting to note, when His Majesty King George III attained his regnal jubilee. Among the objects to which the "Jubilee Fund" will be devoted are, the provision and maintenance of a nursing institute for training nurses both for the hospital and the general public, the establishment of a children's ward, and the improvement of the out-patient department. Already the Fund amounts to over £2,000.

THE Mayor of Bath regards it as a duty for the Chief Magistrate of a city to do his utmost in support of its charities; Mr. Murch not only preaches, but practises. Whenever a local charity requires his assistance, he is ever ready to give it. The meed of praise which he bestowed upon the Eastern Dispensary at the annual meeting last week, was fully deserved. It is one of the oldest and best managed institutions of Bath. Last year no less than 4,668 patients were treated. The income was £543 7*s.* 2*d.*, and a balance in hand £80 9*s.* 10*d.* remained at the end of the year.

---

THE following sums have been recently received by the various Medical Charities, the name of the donor or the source from which they were obtained being given in each instance after the name of the Institution:—

**Legacies.**

Essex and Colchester General Hospital. Mrs. Elizabeth Fisher. £100.
West End Hospital for Nervous Diseases, Welbeck Street Mrs. Bradbury. £500.

**Proceeds of Entertainments.**

West London Hospital. Mr. Silcock's Concert at the Kensington Town Hall. £100.
St. Mary's Hospital, Manchester. Bazaar. £420.

**Subscriptions.**

British Home for Incurables. Company of Clothworkers. £21. Mr. W. S. T. Sandilands. £10.
West London Hospital. Mercers' Company. £10 10*s.*

**Donations.**

British Home for Incurables. Messrs. Marshall and Snellgrove. £10 10*s.*
Charing Cross Hospital. Mr. Edward Brocklehurst. £10.
Hospital for Diseases of the Throat, Golden Square. Mr. George Sturge. £50.
Belfast Royal Hospital. (Endowment of New Wing for Consumptive Patients). Lady Johnson. £1,000. Mrs. Uprichard. £100.
Essex and Colchester General Hospital. Captain J. A. Ind, £52 10*s.*
North London Hospital. Mrs. Fall. £50.
King's College Hospital. Mr. Mathew Whiting. £1,000 per Sister Sibbald of the Twining Ward.

---

THE Bristol Dispensary, the new premises of which are in course of erection, relieved as many as 7,705 cases last year. The Midwifery Department of this institution is one of its most valuable features; no less than 472 poor women of Bristol were attended at their own homes, not a single death occurring. Mr. Rudge, one of the most skilful surgeons of Bristol, has retired from his duties at the Dispensary, after a connection of sixteen years. Dr. Parker has been appointed to succeed him. It is expected that the new premises will be finished by the end of the year.

---

THE out-patient department is a troublesome hospital puzzle. Of its abuse by persons who are not necessitous we have already spoken, and this very abuse intensifies another evil—the overcrowding of the waiting-halls. The Rev. N. Cornford, in the columns of the *Bristol Times*, complains bitterly of the rule at the local hospital, which enforces attendance at 11 a.m., while patients are often kept waiting till 4 p.m. before they can "see the doctor."

THE ladies of Canterbury have begun in right earnest their work in celebration of Her Majesty's Jubilee. In July next a grand fancy bazaar is to be held in aid of the Kent and Canterbury Hospital, with a view to wiping off the existing debt of £1,000. It is also proposed to start a special subscription list, the object of which will be to raise a fund for the endowment of a wing of the Hospital, or a ward, to be christened the "Victoria Ward."

---

THE Lincoln Hospital Saturday Committee, in anticipation of the Queen's Jubilee, are going to make the collection this year at an earlier date than usual, and have fixed June. In July next there is to be a grand bazaar in aid of the County Hospital, and the Saturday Committee have made the somewhat novel proposal to hold one of the stalls in concert with the Hospital Saturday Committees of the neighbouring towns of Gainsborough, Sleaford, and Horncastle. The Hospital Saturday stall ought, at any rate, to stimulate the others to grand achievements.

---

THE Committee of the Leicester Infirmary are very anxious to erect a suitable memorial to the late Dr. Pearce, who, for many years, ably discharged the duties of honorary physician to the institution. It is proposed that the memorial shall take the form of an entrance porch, with a window on either side, one—if not both—of which they suggest, should be in memory of the deceased physician. About £100 is still required to enable the work to be put in hand.

---

THE Committee of the Birmingham Hospital Sunday Fund, in the distribution of hospital and dispensary tickets, appear to us to work on a method which is altogether wrong. The greater the church collection, the more numerous are the tickets consigned to the minister for distribution. Now, it is very evident that the largest contributions will naturally come from the wealthiest congregations. To give the largest supplies of tickets to such churches as these, is something like sending coals to Newcastle. Moreover, the poor, who want them most, will find them the hardest to procure. The Rev. Percival Wilson, rector of All Saints', Birmingham, a parish which has about 30,000 souls, mostly poor, recently received, as his share of the award, *one* ticket, for "distribution among the poor." It is not surprising to learn that the rev. gentleman has been somewhat exercised in his mind as to how he is to "distribute" one ticket among 30,000 persons! To judge the poor man's mite at its intrinsic value, and to award the tickets in mathematical proportion to the sums received from the churches, is simply to pervert the intentions of the donors, and to inflict serious wrong upon the poor.

---

A PROPOSAL has been made to celebrate the Queen's Jubilee in Cambridge by raising a fund which shall form a permanent increase to the endowment of Addenbrooke's Hospital. This old institution, which was founded as far back as 1719, has done, and is doing, an excellent work, but latterly it has had to draw upon its permanent resources to meet the current liabilities.

